# Surgical Treatment of Saphenous Nerve Injury Assisted by Plasma Rich in Growth Factors (PRGF): Lessons from a Case Report

**DOI:** 10.3390/clinpract13050097

**Published:** 2023-09-06

**Authors:** Homid Fahandezh-Saddi Díaz, Antonio Ríos Luna, Manuel Villanueva Martínez, María Elena Cantero Yubero, Roberto Prado, Sabino Padilla, Eduardo Anitua

**Affiliations:** 1 Hand and Upper Extremity Surgery Unit, Hospital Universitario Fundación Alcorcón, 28922 Alcorcón, Spain; 2 Unit for Ultrasound-Guided Surgery, Hospital Beata María Ana, 28007 Madrid, Spain; mvillanuevam@gmail.com; 3 Department of Traumatology and Orthopedic Surgery, Avanfi Institute, 28015 Madrid, Spain; 4 Department of Traumatology and Orthopedic Surgery, Clínica Orthoindal, 04004 Almería, Spain; antonioriosluna@gmail.com; 5 GP Doctor, Área Oeste, CL Arroyomolinos, 28939 Madrid, Spain; elenacy@hotmail.com; 6 Regenerative Medicine Laboratory, BTI—Biotechnology Institute I MAS D, 01007 Vitoria, Spain; roberto.prado@bti-implant.es (R.P.); sabino.padilla1958@gmail.com (S.P.); eduardo@fundacioneduardoanitua.org (E.A.)

**Keywords:** saphenous nerve, surgery, pain, platelet-rich plasma, PRGF

## Abstract

The infrapatellar branch of the saphenous nerve (SN) is a widely described anatomic and functional structure; however, its relevance in daily clinical practice is underestimated. All surgical procedures performed on the anteromedial aspect of the knee are associated with a risk of iatrogenic injury to this nerve, including knee arthroscopy, knee arthroplasty, tibial nailing, etc. We present the case of a saphenous nerve neuroma after treatment with radiofrequency thermal ablation due to a knee pain problem. After conducting an anaesthetic suppression test, we decided to perform a denervation of the medial saphenous nerve in Hunter’s canal. We performed surgery on the anteromedial aspect of the knee. The distal end of the medial SN was coagulated with a bipolar scalpel. The proximal end of the nerve was released proximally, and a termino-lateral suture was made at the free end of the nerve after creating an epineural window to inhibit its growth. A double crush was produced proximally to the suture site to create a grade II-III axonal injury. Autologous plasma rich in growth factors (PRGF) was used to reduce potential post-surgical adhesions and to stimulate regeneration of the surgical lesions. One year after surgery, the patient was living a completely normal life.

## 1. Introduction

The saphenous nerve (SN), which is the terminal sensory branch of the femoral nerve, arises from the femoral nerve at the proximal thigh. It courses through the adductor tunnel alongside the femoral artery and emerges from the tunnel with the descending genicular artery [1,2,3,4]. It splits into the infrapatellar branch, which provides innervation to the proximal tibia inferior and medial to the patella, and the sartorial branch, which travels down the tibial border to supply sensory innervation to the medial aspect of the leg and ankle. The great saphenous vein travels alongside the sartorial branch below the knee joint [1,2,3,4]. This infrapatellar branch of the saphenous nerve (IBSN), after piercing the sartorius muscle, runs with a superficial course and generally forms two branches. Both branches cross the patellar tendon in a transverse way to form the infrapatellar plexus. These small superficial branches are at risk of transection, especially when longitudinal surgical incisions are performed [5,6].

All surgical procedures carried out on the anteromedial aspect of the knee are associated with a risk of iatrogenic injury to this nerve [4]. This can generate numbness in the area innervated by the nerve, neuropathic pain, and the formation of a neuroma. After total knee arthroplasty, numbness due to damage to the IBSN has been reported in 55% to 100% of patients when a longitudinal incision was used [7,8]. Damage to the IBSN has also been reported after surgical meniscectomy (up to 28% of patients report irritating paresthesia), after arthroscopy, after anterior cruciate ligament reconstruction (sensory disturbance was found in 37% to 86% of patients), and even after resections of the prepatellar bursa [6,9,10,11,12,13].

Although only a few authors have mentioned injury to the infrapatellar nerve during tibial nailing, it may contribute to chronic anterior knee pain and is a common and disabling complication in both tibial nailing (10% to 86%) and retrograde femoral nailing (26%) [14,15,16].

Considering these aspects, we describe the case of a saphenous nerve neuroma after treatment with radiofrequency thermal ablation due to a knee pain problem. The patient underwent surgical treatment with denervation, an end-to-side suture to itself, along with double crush and burial in adjacent healthy muscle tissues. A biological approach was used by applying autologous plasma rich in growth factors (PRGF) as an adjunct to surgery to both reduce potential postoperative adhesions and stimulate regeneration of the surgical lesions.

## 2. Case Report

### 2.1. Patient Presentation

This case report is reported according to the CARE (CAse REport) guidelines (Table S1) [17].

A 34-year-old man suffered a motorbike accident in 2016, with an abrasion on the medial aspect of his left knee. Radiographs showed no fracture. After this accident, the patient developed severe, debilitating pain that increased in intensity over the weeks, did not respond to standard analgesics, and required the use of crutches and a wheelchair. Initially, the pain was located in the medial aspect of his left knee, referred to as a burning pain radiating down the medial thigh and leg, which prevented him from resting at night. He was treated with metamizole, corticosteroids, tramadol and pregabalin, with no relief from his pain; he even smoked marijuana in an attempt to control it. After several clinical and imaging tests (X-rays and MRI scans of his left knee did not show signs of injury), a neurophysiological study was performed, which indicated a mild degree of medial saphenous nerve (SN) neuropathy at the level of the medial epicondyle of the knee. The patient underwent rehabilitation with no clinical improvement, and was treated in the pain unit with multiple ultrasound (US)-guided lidocaine infiltrations of the internal SN. The initial response was positive but short-lived. The patient was then treated with 2–3 sessions of pulsed radiofrequency. The pain was partially relieved, but not completely, and later it returned in a more severe form. Though the pain was initially located in the medial aspect of the knee, it later began to radiate proximally in the area of the adductor canal and proximally towards the inguinal ligament. Distally, the pain radiated towards the ankle. At the time of consultation, the patient was a candidate for neurostimulator placement.

### 2.2. Diagnostics

**Figure 1:**
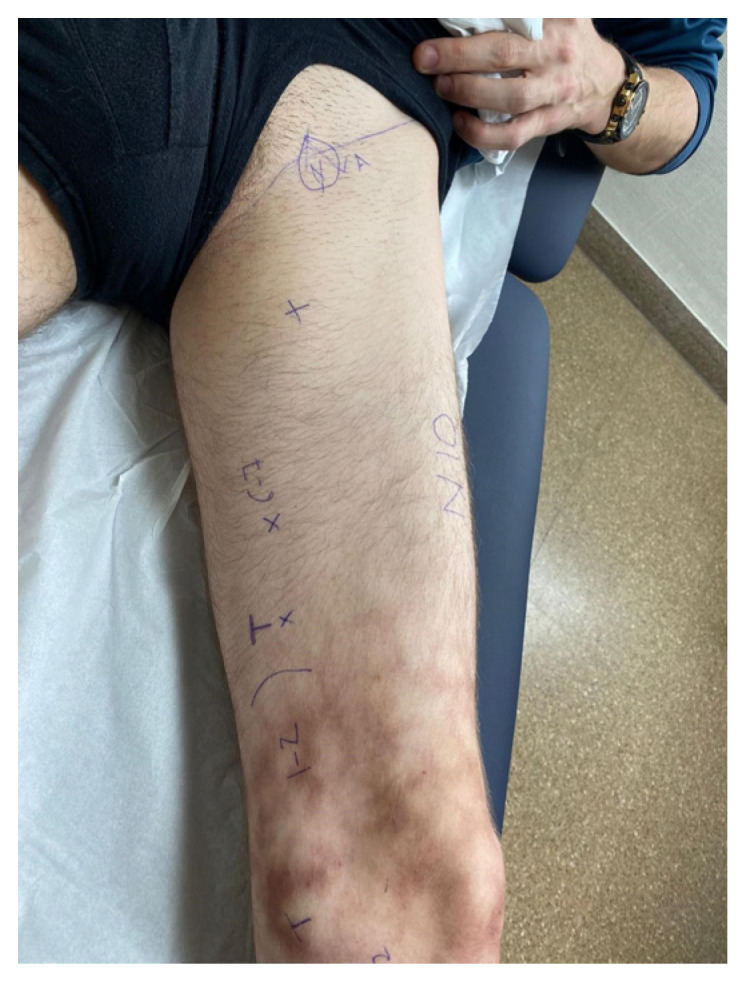
View of the left lower limb. Hypoesthesia in the area of innervation of the medial saphenous nerve in the medial aspect of the knee, proximal leg and distal to the adductor canal. Initial Tinel at the level of the medial joint line of the knee and secondary Tinel around the adductor canal and finally in the inguinal area.

In March 2022, the patient came to our clinic for an initial consultation using crutches due to the intensity of the pain and functional limitation. He had difficulty walking, as full foot support increased the pain. The patient reported that he had not left his home for over a year and that the pain was unbearable. Examination revealed severe atrophy of the entire left thigh’s quadricipital muscle. The patient presented skin burns on the medial aspect of his leg, secondary to the use of high-temperature heating blankets and associated with hyposensitivity in the medial aspect of the knee and down the leg to the ankle. There was also a positive Tinel’s sign in the medial area at the joint level, around Hunter’s canal, and in the groin region. The remainder of the neurological examination was normal, with no signs of femoral nerve compression. The ten-test showed hyposensitivity in the medial saphenous nerve with 1–2/10 (Figure 1).

**Figure 2:**
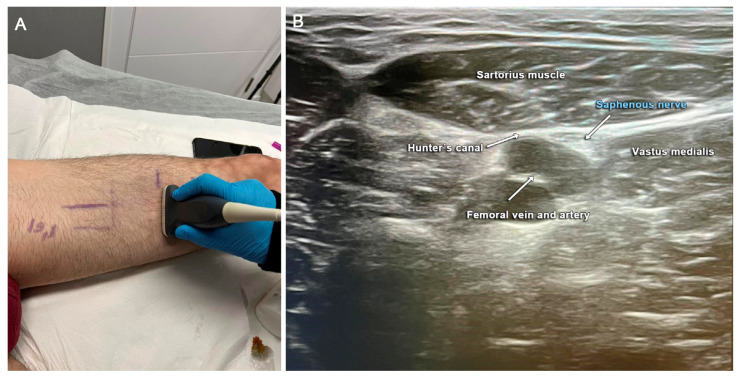
Preoperative images of the case. (**A**) To obtain ultrasound (US) images, the transducer was placed horizontally on the proximal medial thigh to target the adductor canal. (**B**) US image showing how the canal is bordered by the sartorius muscle superficially, the vastus medialis muscle laterally, and the adductor longus muscle deeply. The saphenous nerve appears as a hyperechoic oval structure situated inside the canal with the femoral artery and vein.

With the diagnosis of suspected medial saphenous nerve injury at the level of the joint line and secondary compression in Hunter’s canal and the inguinal ligament due to secondary proximal nerve oedema, a US-guided anaesthetic block test was performed with mepivacaine. By using a US scanner (Alpinion XCube 70) with linear probe SL3-19X, we identified the medial saphenous nerve anterior right medial to the femoral artery, and just distal to the adductor canal (subsartorially). We carried out a suppression test using 2 mL of 1% mepivacaine. The pain response (Figure 2) was assessed after 10 min with a complete resolution of pain in the groin, Hunter’s canal area, and joint line. The patient was able to mobilise the hip and knee and even walk without the aid of a crutch.

### 2.3. Surgical Technique

**Figure 3:**
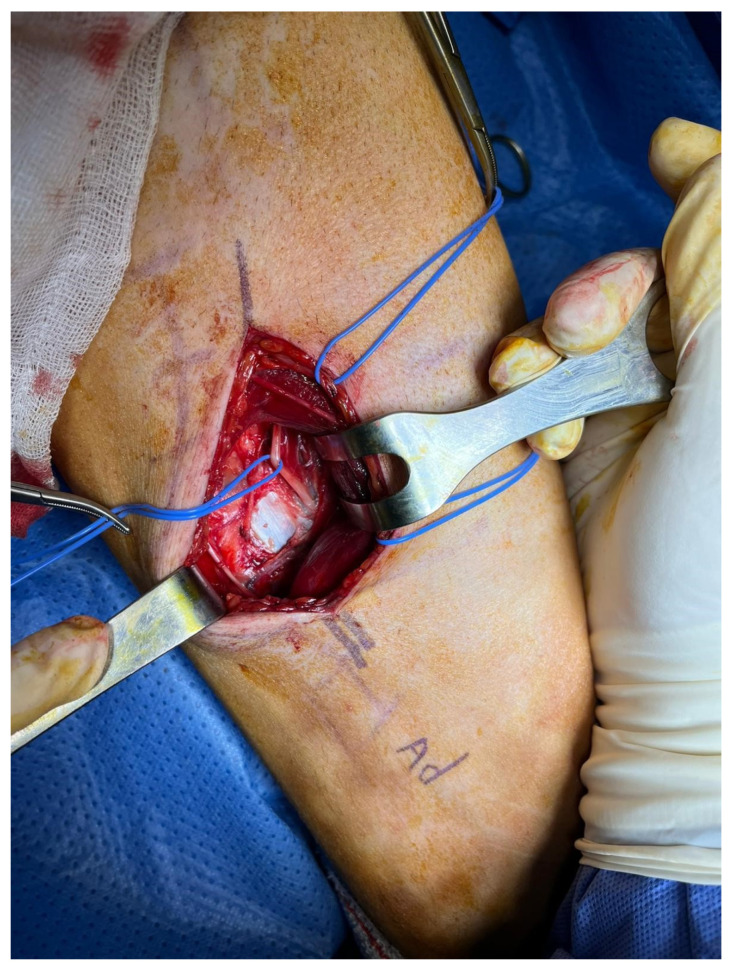
Intraoperative image after separation of the sartorius muscle to the anterior aspect. The saphenous nerve was identified in relation to the femoral artery. This and the femoral bundle are visualised entering the adductor canal.

**Figure 4:**
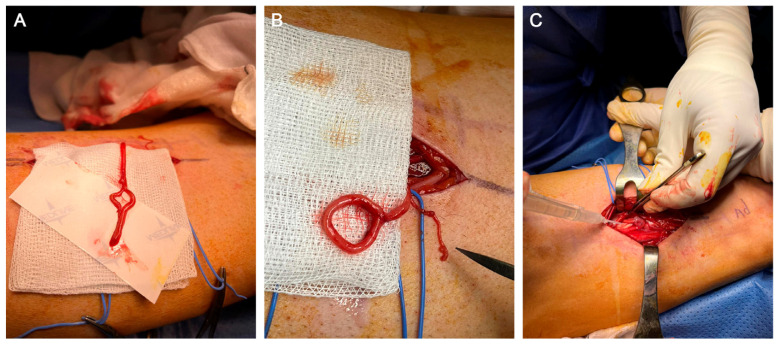
Images of the surgical procedure showing (**A**) the saphenous nerve (SN) after sectioning as distally as possible after opening the adductor canal. (**B**) SN after performing an epineural window and end-to-side neurorrhaphy at 3 cm from its free end. (**C**) Placement of fibrin glue in the suture area of the SN.

**Figure 5:**
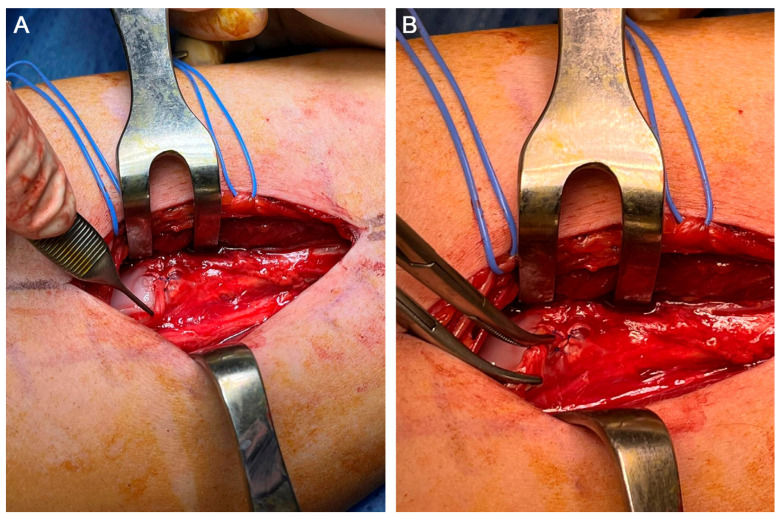
Photographs of the surgery showing (**A**) the burial of the saphenous nerve (SN) in the adjacent medial vastus medialis muscle without tension and (**B**) the proximal double crush of the SN.

Given the obvious response to the anaesthetic suppression test, we decided to perform a denervation of the medial saphenous nerve in Hunter’s canal. The incision was centred using US to reduce the surgical morbidity of the approach. The patient was placed in the supine position without an ischaemic cuff and general anaesthesia was administered due to the patient’s severe pain and anxiety. The incision site was infiltrated with 30 mL of lidocaine 1% with adrenaline 1/100,000. Twenty minutes after the infiltration, a longitudinal incision was made, centred on the area of Hunter’s canal which was previously identified under US guidance. A dissection of the subcutaneous cellular tissue was performed, protecting the sensitive cutaneous branches. Next, the fascia was exposed and the sartorius muscle was identified by delineating the anterior and posterior borders of the sartorius muscle and elevating it from posterior to anterior. At this point, both the femoral artery and vein were identified and in the anterior and medial aspect of the femoral artery, we found the medial saphenous nerve running parallel to the artery and entering Hunter’s canal. We cut through Hunter’s canal and identify the medial saphenous nerve, which divided into its two branches (anterior and posterior) (Figure 3). As the lesion of the medial saphenous nerve was in the medial aspect of the knee joint, we dissected as distally as possible to the nerve. The distal end of the medial saphenous nerve was coagulated with a bipolar scalpel. The proximal end of the nerve was released proximally, and a termino-lateral suture was made at the free end of the nerve after creating an epineural window to inhibit its growth. The suture area was then reinforced with fibrin glue (Tissucol Duo) (Figure 4A–C). Next, a double crush was produced proximally to the suture site to create a grade II-III axonal injury. A small buttonhole was made in the area of the vastus medialis muscle to bury the end of the saphenous nerve where the omega (termino-lateral) suture was made (Figure 5A,B).

### 2.4. Plasma Rich in Growth Factors (PRGF) Preparation and Application

**Figure 6:**
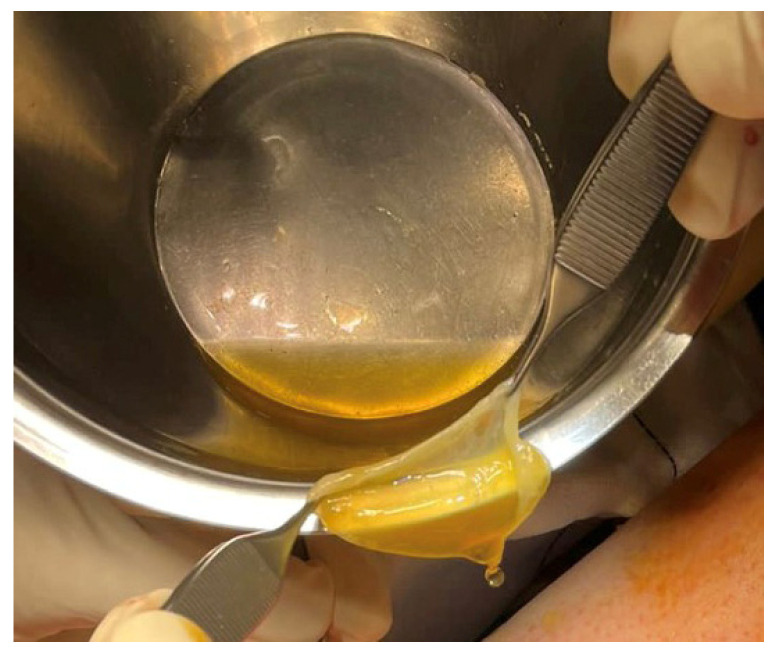
Preparation of a plasma rich in growth factors (PRGF) membrane to wrap the saphenous nerve proximally.

Autologous PRGF was used to reduce potential post-surgical adhesions and to stimulate regeneration of the surgical lesions. All procedures were performed under sterile conditions in the operating theatre and according to Anitua et al. [18]. Briefly, 72 mL of peripheral venous blood was collected in 9 mL tubes containing 3.8% (wt/vol) sodium citrate (EDK2_ENV kit, BTI Biotechnology Institute, S.L., Vitoria, Spain). The blood was centrifuged at 580× *g* for 8 min at room temperature. The upper plasma fraction (F1), containing a platelet count similar to that of peripheral blood, was collected and stored for posterior infiltration. The 2 mL plasma fraction (F2), located just above the sedimented red cells but without the buffy coat, was collected in another collection tube for membrane preparation. This PRGF F2 contained a moderate enrichment of platelets (approximately 2 times the platelet count of peripheral blood) without leukocytes. To generate PRGF membranes, 8 mL of PRGF F2 was activated with calcium chloride (10% wt/vol) and incubated at 37 °C for 20–30 min to allow the formation of a biocompatible fibrin membrane. One PRGF fibrin membrane was placed around the saphenous nerve after distal sectioning and termino-lateral anastomosis, and a second membrane was placed in the buttonhole created in the sartorius muscle (Figure 6). Finally, the sartorius area was closed with Vicryl 2/0, Vicryl 3/0 for the subcutaneous cellular tissue and Ethilon 4/0 at skin level. In order to improve wound healing and reduce post-surgical pain, freshly activated PRGF F1 was infiltrated subcutaneously.

### 2.5. Postoperative and Evolution

The patient walked with the aid of two crutches for 10 days, which were progressively abandoned, and at 3 weeks, the patient walked without any assistance. One year after surgery, the patient was living a completely normal life, without pain and without taking painkillers. He is now involved in sports and has returned to work as a security guard.

## 3. Discussion

The saphenous nerve (SN) is a sensory nerve branch of the femoral nerve that provides innervation from the medial thigh to the medial knee and great toe. A major branch of the saphenous nerve is the infrapatellar nerve, which arises at the medial knee. The specific location and anatomy of this nerve make it susceptible to damage from falls (contusion), as in our patient, and especially during knee surgery [1,2,3,4,5]. The SN has variations in its topographic anatomy, especially affecting the infrapatellar branch.

Kerver et al. in 2013 [4] showed that the IBSN is at risk of iatrogenic damage in any surgery on the anteromedial aspect of the knee, especially when longitudinal incisions are used. Three low-risk zones of iatrogenic nerve injury were identified: one was located on the medial side of the knee, at the level of the tibial tuberosity, in which a −45° oblique incision was least prone to damaging the infrapatellar branch, and the other two zones were located medial to the patellar apex (cranial and caudal), in both of which nearly horizontal incisions were least prone to injuring the infrapatellar branch. In order to minimise iatrogenic damage to the IBSN and whenever technically possible, the direction of the incisions should be parallel to the direction of the nerve [4]. This condition is relatively uncommon, accounting for <1% of adult patients with lower extremity pain [4]. This may be due to being underdiagnosed [4,7,8]. Iatrogenic injury to this branch has been documented since 1945 as a potential complication after medial arthrotomy [19]. Neuritis of the IBSN can also result from entrapment, bursitis, or patellar dislocation [20].

Moreover, injury of IBSN is a possible complication after knee surgeries [6,9,10,11,12,13,16] and radiofrequency therapies. We can find injuries of IBSN in arthroscopic surgeries because of the proximity of the saphenous nerve and branches to surgical site. Mochida and Kikuchi [21] found that 22.2% of patients experienced sensory disorders in the IBSN after knee arthroscopy. Another setting for IBSN injury is following ACL reconstruction, especially when a hamstring graft is harvested. Other authors reported that IBSN injury incidence is higher in patellar tendon autologous harvesting, while no nerve injury was reported when a quadricep tendon was used [22]. IBSN injury during intramedullary tibial nailing was also described [14,15,16]. For example, Shi et al. [16] reported that the nerve was injured when conducting this procedure at multiple sites, including the medial parapatellar approach, where the medial retinacular layer was directly lacerated; proximal medial-to-lateral locking screw placement, which resulted in either direct screw injury to the nerve or entrapment of the fascia covering the nerve; direct scalpel incision in the infrapatellar region; and entrapment caused by the closure of successive layers in the infrapatellar incision. Finally, SN injury is the most common complication after surgical treatment of varicose veins [1]. The close proximity of the vein and nerve, particularly around the shank region, during vein resection procedures often leads to nerve damage, especially in patients with advanced varicose veins in this area due to numerous insufficient perforators.

Therefore, when we perform a surgery on the anteromedial aspect of the knee, we have to pay attention to the IBSN, and when we perform a surgery on the middle aspect of the leg close to the saphenous vein, the sartorial branch must be identified, dissected, and protected [1,2,3,4]. After the surgery, careless dissection or a lack of knowledge of SN or its branches will lead to sensory alterations of this sensory nerve, including sensory loss, pain, paraesthesia, and anaesthesia in the middle aspect of the leg. Sometimes, a painful neuroma can be found, associated with a Tinel sign [8,13,19]. Although, electrophysiological studies can help in the diagnosis [23], as in our case, clinical and neurological examination are the most important assessments for the diagnosis of injury or entrapment of the SN and its branches. In addition, a US-guided anaesthetic suppression test is pivotal in severe neuropathic pain with a suspicion of nerve entrapment or nerve injury. It is important to point out that there is a great deal of anatomical variability in the area of the adductor canal [24]. In fact, Saranteas et al. [25] showed in a dissection of 11 specimens how the vastoadductor membrane is a thickened fascia that overlies the SN. In 82% of cases (9 specimens), this nerve ran through and exited the adductor canal distally, whereas in two cases, it crossed the vastoadductor membrane more proximally. Furthermore, in one case, the SN was formed by the union of two branches, with one entering the adductor canal, and a second branch originating from the femoral nerve, parallel to the femoral artery but outside the adductor canal [25]. Knowing this anatomical variability, we perform the anaesthetic suppression test as described by Saranteas et al. [25]. The safest way to perform the US-guided anaesthetic block is to identify the SN in the area distal to the adductor canal, where the SN lies between the sartorius muscle and the femoral artery. In our case, we did not use a stimulator to perform the block, and only introduced 2 mL of 1% mepivacaine under US guidance [26] into the site.

US-guided anaesthetic block allows one to identify the nerve lesion and block conduction, thereby eliminating pain and allowing correct surgical planning [24,25,27]. In our case, by blocking the SN at Hunter’s canal, the referred pain and Tinel’s sign disappeared proximally and distally, indicating that the SN denervation should be performed at the level of the adductor canal. Therefore, releasing the SN proximal to the inguinal ligament should be avoided. Moreover, this situation had a positive prognostic effect, because if we eliminate the pain with a small amount of anaesthesia, we will probably relieve the patient’s symptoms with an operation on this nerve, whether it is a release or a denervation.

Conservative management of neuropathic pain includes several types of treatment, such as oral or topical analgesics, the modification of activities of daily living, transcutaneous electrical stimulation, nerve blocks with corticosteroids and local anaesthetics, cryoneuroablation, pulsed and continuous radiofrequency [28,29], the application of particulate amniotic umbilical cord [30], and the use of platelet-rich plasma (PRP) [31,32,33,34], among others. The PRP we used was PRGF, which has a moderate concentration of platelets and is free of leucocytes and erythrocytes, and so it can be classified as a pure P-PRP [35]. The aim of wrapping the nerve with the PRGF membrane was two-fold. On the one hand, it helped to avoid surgical adhesions, and on the other hand, it helped to stimulate regeneration with an extra supply of growth factors and other bioactive molecules involved in nerve regeneration, such as NGF, BDNF, IGF-1, PDGF, VEGF, HGF, TGF-β, fibronectin and vitronectin [32,34,36].

However, neuropathic pain often persists and requires surgical treatment. In the case of pure nerve entrapment, a medial saphenous nerve release is performed at the level of the affected area, usually in the adductor canal, as described by Dellon and Mackinnon [37] with positive outcomes. In the case of a saphenous nerve neuroma, various techniques have been described. Schur et al. [38] presented nine cases of saphenous nerve neuromas after knee arthroscopy in which the neuroma was resected, sutured to a nerve allograft and the free end embedded in healthy muscle. Six of the nine patients reported subjective improvement through final follow-up. Three of the nine patients reported initial improvement, with recurrence of pain at/near the site of the neuroma. Other authors [37,39] resected the neuroma, coagulated its end with a bipolar scalpel, performed a double crush to reproduce a grade II-III axonotmesis, and finally buried the nerve in healthy muscle or bone. Conversely, Aszmann et al. [40] advocated end-to-side neurorrhaphy into adjacent sensory and/or motor nerves to provide a target organ for axons. These authors presented a series of 16 cases and achieved clinical improvement in 15 out of 16 patients. In cases where the saphenous nerve has a single fascicle at the level of the adductor canal, as in our case, we can perform this end-to-side neurorrhaphy technique by associating a proximal double crush and burying the nerve without tension in an adjacent healthy muscle. In the case where there are two fascicles of the saphenous nerve, we can carry out an end-to-end suture of both fascicles, a double crush, and a relocation in an adjacent healthy muscle.

## 4. Conclusions

Medial saphenous nerve injuries are rare and often underdiagnosed. It is important to identify and protect the infrapatellar branch of the saphenous nerve after surgery or procedures performed in the antero-medial aspect of the knee. Burning pain, hypoesthesia and Tinel’s sign in the area innervated by the saphenous nerve must be excluded after surgery in the antero-medial knee area, and the clinical history and examination are key to diagnosis. When the diagnosis is suspected, a US-guided anaesthetic suppression test of the saphenous nerve at the level of the adductor canal is a reliable test that provides a definitive diagnosis in a fast and simple way. If conservative treatment fails, surgical treatment via denervation, an end-to-side suture to itself, adding a double crush and burial in adjacent healthy muscle is a safe technique with good results. The use of PRGF as an adjunct to surgery reduced both the risk of adhesions and post-operative pain and inflammation.

## Data Availability

The data supporting the findings in this case report are available within the article.

## Supplementary Materials

The following supporting information can be downloaded at: https://www.mdpi.com/article/10.3390/clinpract13050097/s1, Table S1: Checklist of CARE (CAse REport) guidelines.

## Abbreviations

IBSN	Infrapatellar branch of the saphenous nerve
PRGF	Plasma rich in growth factors
PRP	Platelet-rich plasma
SN	Saphenous nerve
US	Ultrasound
