# Supplementary material for: Surgical Treatment of Saphenous Nerve Injury Assisted by Plasma Rich in Growth Factors (PRGF): Lessons from a Case Report
Source: Clin Pract. 2023 Sep 6;13(5):1090–9. doi: 10.3390/clinpract13050097 (PMC10514825; doi:10.3390/clinpract13050097)
Supplement: Supplementary file 1 [file clinpract-13-00097-s001.zip › clinpract-2544496-supplementary.pdf]

Reporting checklist for case report or case series.

Based on the CARE guidelines.

Instructions to authors

Complete this checklist by entering the page numbers from your manuscript where readers will find each of the items listed below.

Your article may not currently address all the items on the checklist. Please modify your text to include the missing information. If you are certain that an item does not apply, please write "n/a" and provide a short explanation.

Upload your completed checklist as an extra file when you submit to a journal.

In your methods section, say that you used the CAREreporting guidelines, and cite them as:

Gagnier JJ, Kienle G, Altman DG, Moher D, Sox H, Riley D; the CARE Group. The CARE Guidelines: Consensus-based Clinical Case Reporting Guideline Development

		Reporting Item	Page Number
**Title**			
	#1	The area of focus and "case report" should appear in the title	1
**Keywords**			
	#2	Two to five key words that identify topics in this case report	1
**Abstract**			
Introduction	#3a	What is unique and why is it important?	1
	#3b	The patient's main concerns and important clinical findings.	1
	#3c	The main diagnoses, interventions, and outcomes.	1
Conclusion	#3d	What are one or more "take-away" lessons?	1
**Introduction**			

	#4	Briefly summarize why this case is unique with medical literature references.	1-2
**Patient information**			
	#5a	De-identified demographic and other patient information.	2
	#5b	Main concerns and symptoms of the patient.	2-3
	#5c	Medical, family, and psychosocial history including genetic information.	2
	#5d	Relevant past interventions and their outcomes.	2-3
**Clinical findings**			
	#6	Relevant physical examination (PE) and other clinical findings.	2-3
**Timeline**			
	#7	Relevant data from this episode of care organized as a timeline (figure or table).	2-3
**Diagnostic assessment**			
	#8a	Diagnostic methods (PE, laboratory testing, imaging, surveys).	2-3
	#8b	Diagnostic challenges.	2-3
	#8c	Diagnostic reasoning including differential diagnosis	2-3
	#8d	Prognostic characteristics when applicable	2-3
**Therapeutic Intervention**			
	#9a	Types of intervention (pharmacologic, surgical, preventive).	3-5
	#9b	Administration of intervention (dosage, strength, duration)	3-5
	#9c	Changes in the interventions with explanations.	3-5

**Follow up and outcomes**			
	#10a	Clinician and patient-assessed outcomes when appropriate	6
	#10b	Important follow-up diagnostic and other test results.	6
	#10c	Intervention adherence and tolerability (how was this assessed)?	6
	#10d	Adverse and unanticipated events.	N/A
**Discussion**			
	#11a	Strengths and limitations in your approach to this case.	6-8
	#11b	Discussion of the relevant medical literature.	6-8
	#11c	The rationale for your conclusions.	8
	#11d	The primary “take-away” lessons from this case report.	8
**Patient perspective**			
	#12	The patient can share their perspective on their case	N/A
**Informed consent**			
	#13	The patient should give informed consent.	9

None The CARE checklist is distributed under the terms of the Creative Commons Attribution License CC-BY-NC. This checklist can be completed online using https://www.goodreports.org/, a tool made by the EQUATOR Network in collaboration with Penelope.ai
